# Neurological manifestations in COVID-19 and its possible mechanism

**DOI:** 10.18632/aging.103732

**Published:** 2020-09-27

**Authors:** Xiaojia Tang, Yuhan Luo, Yuxia Song, Hongyang Fan, Sisi Dong, Peipei Liu, Yingzhu Chen

**Affiliations:** 1 Department of Neurology, Dalian Medical University, Dalian 116000, Liaoning, China; 2 Department of Neurology, Clinical Medical College, Yangzhou University, Yangzhou 225001, Jiangsu, China; 3 The Second Xiangya Hospital, Central South University, Changsha 410000, Hunan Province, China

**Keywords:** COVID-19, SARS-CoV-2, polyneuropathy, rhabdomyolysis, cerebrovascular disease

## Abstract

In December 2019, the first cases of the acute respiratory illness now known as Corona Virus Disease 2019 (COVID-19) occurred in Wuhan, Hubei Province, China. The main clinical manifestations of COVID-19 are a fever, dry cough and general weakness, although in some patients, a headache, tight chest, diarrhea, etc. are the first clinical manifestations. Neurological practice is involved in all aspects of medicine, from primary care for patients with migraines to consultations with patients in the intensive care unit. Few disorders spare the nervous system, and newly emerging infections are no exception. As neurologists, we are concerned about the effects of SARS-CoV-2 infections on the nervous system. Multiple neuropathy, rhabdomyolysis, cerebrovascular disease, central nervous system infections and other common neurological diseases require attention during this outbreak.

## INTRODUCTION

In December 2019, a number of unexplained cases of pneumonia occurred in Wuhan, China, and rapidly spread to other parts of China, then to Europe, North America, Asia and most of the world. This outbreak was confirmed to be caused by a novel coronavirus – severe acute respiratory syndrome coronavirus 2 (SARS-CoV-2) [1, 2]. On March 11, 2020, the World Health Organization declared Corona Virus Disease 2019 (COVID-19) as a pandemic [3]. As of May 7, 2020, there were 3,672,238 confirmed cases of COVID-19 and 254,045 deaths due to the disease globally [4]. The most common symptoms in patients diagnosed with SARS-CoV-2 infections are a fever and dry cough [5, 6]. Infections caused by SARS-CoV-2 exhibit many clinical similarities to those caused by SARS-CoV, such as a fever, dry cough and diarrhea during the prodromal phase [7–9].

In addition to the typical respiratory symptoms, some SARS patients have had neurological problems [10, 11]. Similarly, in some patients diagnosed with COVID-19, headaches, muscle aches, confusion and seizures have been the first clinical manifestations [12, 13]. In COVID-19 epidemic areas, people who have had close contact with diagnosed COVID-19 patients should be alert to the possibility of SARS-CoV-2 infection if they develop neurological symptoms such as headaches, slurred speech, hemiplegia and disturbances of consciousness. This article reviews the epidemiology of SARS-CoV-2 infections, the neurological diseases related to SARS-CoV-2, and the possible mechanisms behind these relationships.

### SARS-CoV-2

SARS-CoV-2, originally named 2019-nCoV, belongs to the broader family of coronaviruses [1, 2]. Coronaviruses are enveloped, positive-stranded RNA viruses that belong to the family Coronaviridae and the order Nidovirales. Six coronavirus species are known to cause respiratory, enteric, hepatic and neurologic diseases. Four of these viruses are prevalent – 229E, OC43, NL63 and HKU1 – and typically cause common cold symptoms in immunocompetent individuals [14]. The two other strains –SARS-CoV [15] and Middle East respiratory syndrome coronavirus (MERS-CoV) [16] – are zoonotic in origin, and have been linked to sometimes fatal illness. SARS-CoV-2 is the seventh member of the coronavirus family. Zhou et al. found that SARS-CoV-2 was 96% identical at the whole-genome level to a bat coronavirus, so SARS-CoV-2 may have originated in bats [17].

An epidemic or outbreak can occur when the agent (pathogen), population (hosts) and environment create an ideal situation for spread [18]. The current evidence suggests that SARS-CoV-2 may have spread to humans via wild animals sold illegally in the Huanan Seafood Wholesale Market [19]. The extent of human-to-human transmission of SARS-CoV-2 was unclear at first, but now there is evidence of human-to-human transmission [5, 20, 21]. The main sources of infection are SARS-CoV-2-infected patients, including those who are asymptomatic [22]. The routes of transmission include droplet transmission, contact transmission and aerosol transmission [23, 24]. In a recent study, SARS-CoV-2 was detected in stool samples from patients with abdominal symptoms [20, 25], so some scholars have proposed that SARS-CoV-2 could spread *via* fecal-oral transmission. Further environmental studies will be needed to determine whether the virus remains viable under conditions that would favor fecal-oral transmission [26]. SARS-CoV-2 has not been confirmed to be transmitted vertically from mother to child [27]. Based on the available data, a Chinese team estimated a basic reproduction number (R0) of 3.77 for SARS-CoV-2, basically confirming that the new coronavirus is more contagious than SARS [28].

SARS-CoV-2 employs a densely glycosylated, homotrimeric class I fusion spike (S) protein to enter host cells. The S protein exists in a metastable prefusion conformation that undergoes a dramatic structural rearrangement to fuse the viral membrane with the host cell membrane [29, 30]. Epidemiological data indicate that the population is generally susceptible to SARS-CoV-2 [31]. Therefore, it is necessary for individuals to wash or disinfect their hands frequently, go outside less, wear a mask, avoid group activities, stay away from patients with COVID-19, maintain good living habits and keep an optimistic attitude [32–34].

### Neurological disease

SARS-CoV-2 has been reported to be associated with Guillain-Barré syndrome, rhabdomyolysis, acute cerebrovascular disease, central nervous system infections and other neurological diseases. Four formal reports have described neurological problems in SARS patients, including polyneuropathy [35], myopathy and rhabdomyolysis [36], large artery ischemic stroke [37] and central nervous system infections [38]. Human coronaviruses (HCoVs) can naturally reach the central nervous system, and could potentially cause neurological symptoms. Among the coronavirus-induced animal diseases, feline infectious peritonitis virus, mouse hepatitis virus and hemagglutinating encephalomyelitis virus can all reach the central nervous system and induce different types of neuropathologies [10, 11]. The structure of SARS-CoV-2 is similar to that of the SARS virus, and both viruses invade the human body through the angiotensin converting enzyme II (ACE2) receptor. Thus, in this paper, we mainly describe the neurological diseases associated with SARS-CoV-2, but also briefly introduce the neurological diseases associated with SARS.

#### Neuromuscular manifestations

#### Polyneuropathy

Polyneuropathy, also known as peripheral neuropathy, is multiple-nerve damage of the extremities. The clinical manifestations are mostly distal symmetrical motor sensory dysfunction and autonomic nerve dysfunction [39]. The causes of polyneuropathic disorders include metabolic, toxic, infectious, inflammatory, autoimmune and genetic conditions [40]. Zhao et al. reported a case of COVID-19 initially presenting with acute Guillain-Barré syndrome (GBS). The female patient aged 61 years presented with acute weakness in both legs and severe fatigue. She received intravenous immunoglobulin, antiviral drugs of arbidol, lopinavir, and ritonavir, and supportive care. After 30 days of treatment, the muscle strength of the limbs returned to normal and the respiratory symptoms disappeared [41]. In a recently published article, two COVID-19 patients were diagnosed with Miller-Fisher syndrome (MFS) and multiple cranial neuritis, respectively [42]. These cases suggest a possible link between GBS and SARS-CoV-2 infection.

Some patients with severe COVID-19 progress rapidly and need to be transferred to an intensive care unit (ICU) for further treatment [43, 44]. In such patients, the peripheral nerves could be particularly susceptible to peripheral microcirculation disturbances, since the vessels supplying them with blood lack autoregulation [45]. ICU-acquired weakness, which can manifest as critical-illness polyneuropathy, critical-illness myopathy or both, is a frequent and disabling disorder in ICU patients [46]. Critical-illness polyneuropathy, an axonal sensory-motor polyneuropathy, is observed in up to a third of critically ill patients with systemic inflammatory response syndrome. Critical-illness myopathy, an acute myopathy, develops in a similar setting, often in association with the use of corticosteroids and/or nondepolarizing neuromuscular-blocking agents [47].

Tsai et al. [35] presented data from four patients with probable SARS who developed axonal polyneuropathy, myopathy or both (2004). All of them had received intubation for respiratory distress and a high dose of steroid therapy for multiple organ failure. They developed distal-predominant weakness in all four limbs and a mild decrease in deep-tendon reflexes three to four weeks after the onset of SARS. The most likely diagnoses were critical-illness polyneuropathy and/or critical-illness myopathy. Some viruses, such as cytomegalovirus and varicella zoster virus, may cause peripheral neuropathy by directly attacking the nerves. It is not known whether direct attacks of the peripheral nervous system occur in HCoV-associated neuropathy.

#### Rhabdomyolysis

Rhabdomyolysis refers to the damage to striated muscle, the destruction of the muscle cell membrane integrity and the release of myoglobin, creatine kinase, other enzymes, small molecules and toxic substances into the systemic circulation due to various traumatic and non-traumatic factors, resulting in a group of clinical syndromes of organ damage [48, 49]. Clinical examination, history evaluation, laboratory studies, muscle biopsies and genetic testing are useful tools for diagnosing rhabdomyolysis and differentiating acquired from inherited cases. Acquired cases may be due to substance abuse, medication or toxic exposures, electrolyte abnormalities, endocrine disturbances and autoimmune myopathies [50].

In several recent studies on COVID-19 [5, 12, 20], a few patients exhibited varying degrees of myalgia, fatigue and elevated creatine and creatine kinase levels. In the study of Guan et al., two patients clearly developed rhabdomyolysis as a complication of COVID-19, while 14.90% (164/1099) exhibited myalgia or arthralgia symptoms and 13.7% (90/657) had creatine kinase levels ≥ 200 U/L [5]. Tong’s research group reported that a patient diagnosed with COVID-19 had pain and weakness in both lower limbs and obvious tenderness after the ninth day of admission. Laboratory examination indicated that the patient’s myoglobin level was >12,000.0 μg/L (reference 0-140 μg/L), creatine kinase was 11,842 U/L (reference 38-174 U/L) and lactate dehydrogenase was 2,347 U/L (reference 109-245 U/L). The authors added hydration, alkalization, plasma transfusion, gamma globulin and symptomatic support therapy based on the patient’s previous treatment with oxygen, antivirals, antibiotics and methylprednisolone [51]. Creatine kinase and myoglobin are important indicators of rhabdomyolysis, but they are not routinely detected in the clinical practice. When patients have local muscle pain and weakness, rhabdomyolysis should be considered.

In previous SARS studies, some patients were clearly diagnosed with critical-illness myopathy [35] and rhabdomyolysis [50, 52]. In such patients, it cannot be ruled out that rhabdomyolysis may have developed due to the use of corticosteroids and/or nondepolarizing neuromuscular-blocking agents; however, the association of rhabdomyolysis with viruses such as influenza viruses A and B, human immunodeficiency virus, Coxsackie virus, cytomegalovirus, West Nile virus and dengue virus has also been well described [53–56]. Nevertheless, there is not yet sufficient evidence that HCoVs can directly invade muscle cells.

#### Acute cerebrovascular disease

The population is generally susceptible to SARS-CoV-2, but the elderly are more susceptible (the median age of hospitalized patients in one study was 56 years [interquartile range, 42-68 years; range, 22-92 years] [20]), and such patients are already at high risk for cerebrovascular diseases. Viral infections are known to be associated with an increased risk of stroke [57]. In a study by Mao et al., 214 patients diagnosed with COVID-19 were enrolled, and six (2.80%) of them developed acute cerebrovascular disease (five cases of ischemic stroke and one case of cerebral hemorrhage). All but one of these patients (an ischemic stroke patient) died of respiratory failure [13]. In a study of 206 SARS patients in Singapore, large artery stroke was diagnosed in five patients, of whom four were critically ill and three died [58]. Strokes are not uncommon in critically ill patients with multiple comorbidities, so SARS-CoV-2 infections in humans may increase the risk of stroke.

#### Central nervous system infection

Central nervous system infections are among the most critical problems in public health, as patients frequently exhibit neurologic sequelae. The clinical manifestations include a fever, headache, vomiting, stiff neck, afebrile seizures and status epilepticus. HCoVs cause a certain degree of nerve erosion, but their capacity to infect the central nervous system in humans has not been well characterized [10, 59]. Moriguchi et al. described a patient with SARS-CoV-2-associated meningitis who was brought to the hospital by ambulance due to convulsions and a coma. Interestingly, SARS-CoV-2 RNA was not detected in the patient’s nasopharyngeal swab, but was detected in the patient’s cerebrospinal fluid [60]. Zhao et al. [61] reported spinal cord involvement in a COVID-19 patient one week after the onset of fever. After admission, his SARS-CoV-2 RNA nasopharyngeal swab test was positive. Based on the patient’s acute flaccid myelitis of the lower limbs, urinary and bowel incontinence, and sensory level at T10, a diagnosis of acute myelitis was more likely. After the patient had been treated with high-flow oxygen, antiviral medication, steroids and human immunoglobulin, his body temperature returned to normal and two subsequent SARS-CoV-2 RNA nasopharyngeal swab tests were negative. The muscle strength of both upper limbs recovered to grade 4/5, while the muscle strength of both lower limbs was grade 1/5. This study indicated that acute myelitis may be a neurological complication of COVID-19. The above cases demonstrate the potential for neurological invasion of SARS-CoV-2.

The presence of HCoV in human central nervous system-related samples was detected as early as 1980 in autopsies of patients with multiple sclerosis [62]. In 2004, genetic material from SARS-CoV was detected in cerebrospinal fluid samples from a 32-year-old woman. The patient had a generalized tonic-clonic convulsions with loss of consciousness and up-rolling eyeballs lasting for one minute [38]. Another patient, a doctor infected with the SARS virus, had symptoms of restlessness, vomiting and confusion on the 33th day of illness. The patient died after treatment failed, and a brain biopsy was performed. A fragment specific for SARS HCoV was amplified from cultures of the brain suspension, and transmission electronic microscopy revealed the presence of an enveloped virus morphologically compatible with a coronavirus in the cultures [63]. Since some COVID-19 patients have complained of headaches, nausea etc, care providers should be alert for central nervous system infections caused by SARS-CoV-2 if such patients also exhibit symptoms such as a fever, epilepsy and disturbances of consciousness.

### Mechanisms of nervous system damage due to SARS-CoV-2 infections

In this section, we will explore various mechanisms that may explain the correlation between COVID-19 and neurological disease.

#### Hypoxemia

In a clinical retrospective study of 138 people, the most common complication of COVID-19 during hospital admission was pneumonia, followed by acute respiratory distress syndrome (19.60%) and shock (8.70%) [20]. The patients in this study had varying degrees of hypoxia, accompanied by hypoxemia. Most patients received oxygen inhalation (ordinary oxygen inhalation, 106 [76.81%]), and many received mechanical ventilation (non-invasive ventilation, 15 [10.09%]; intermittent mandatory ventilation, 17 [12.32%]). More than 20% of the oxygen consumed by humans is used by the brain for ATP production to generate the required membrane potential [64]. As soon as anoxia sets in, ATP synthase begins to pump protons out of the mitochondrial matrix to maintain the mitochondrial membrane potential. Continued lack of oxygen can eventually lead to the loss of high-energy phosphate esters, disturbances of neurotransmitter metabolism, the breakdown of the membrane, the failure of mitochondria and the accumulation of intracellular Ca^2+^. The immediate consequence is irreversible neurological damage and even neuronal death [64, 65].

Lack of oxygen increases the risk of stroke. For instance, the prolonged hypoxia of obstructive sleep apnea hypopnea syndrome can damage the sleep structure, increase blood pressure, reduce cerebral blood flow and promote microthrombosis and atherosclerosis, thus impacting the prognosis and recurrence of cerebral infarction [66, 67]. Mao et al. reported that six COVID-19 patients had acute cerebrovascular disease: five with severe infections (5/88) and one with a non-severe infection (1/126) (P=0.03) [13]. The symptoms of hypoxia in COVID-19 patients are very obvious, and critical patients need ventilator support. COVID-19 patients admitted to the ICU tend to be older and have a greater number of comorbid conditions (e.g., hypertension, diabetes, cardiovascular and cerebrovascular diseases) than those not admitted to the ICU [20]. This suggests that older age and these comorbidities may be risk factors for poor outcomes [68, 69].

#### ACE2

The metallopeptidase ACE2 has been confirmed to be the cell receptor for SARS-CoV-2, just as it is for SARS-CoV [70, 71]. However, SARS-CoV-2 cannot enter cells through other coronavirus receptors such as aminopeptidase N and dipeptidyl peptidase [71]. ACE2 is highly expressed not only in the alveolar type II cells of the lungs and the upper and stratified epithelial cells of the esophagus, but also in the absorptive enterocytes of the ileum and colon [72, 73]. The main physiological function of ACE2 is to catalyze the conversion of angiotensin II to angiotensin (1-7), with a vasodilator effect. In brain tissues, angiotensin (1-7) stimulates Mas receptors to promote angiogenesis, and also inhibits oxidative stress, prevents neuroinflammation, improves cerebral blood flow, suppresses apoptosis and protects cerebral blood vessels [74].

Enhancing the expression of ACE2 may be an important strategy for treating cardiovascular and cerebrovascular diseases [75]. SARS-CoV-2 patients with cerebrovascular disease may be more likely to develop into severe patients with a higher risk of death, so more timely diagnosis is needed for such patients. ACEI and angiotensin II receptor blocker antihypertensive drugs may increase the expression of the ACE2 receptor [76]. In order to avoid aggravating SARS-CoV-2 infection symptoms, it is recommended that hypertensive patients on blood pressure control medications stop using ACEI and angiotensin II receptor blocker antihypertensive drugs, and instead use calcium channel blocker diuretic antihypertensive drugs [77].

#### Immunization

The responses of the immune system can be divided into innate immunity (also known as non-specific immunity) and adaptive immunity (also known as specific immunity, which can be further divided into humoral immunity and cellular immunity) [76, 78]. The immune mechanisms induced by SARS-CoV-2 are unclear. After SARS-CoV-2 enters the body through ACE2, host factors trigger an immune response against the virus. The virus induces natural immunity, phagocytosis and phagocytic cell death, thus damaging tissues and organs. In four clinical retrospective studies that clearly identified the diagnosis of COVID-19 [12, 19, 20, 79], the absolute value of lymphocytes in most patients was reduced. These findings suggest that SARS-CoV-2 mainly attacks lymphocytes, especially T lymphocytes, similar to SARS-CoV.

CD4+ T cells are well known to regulate or “assist” the functioning of other lymphocytes. CD8+ T cells are cytotoxic and can kill virus-infected cells [80]. Barton et al. [81] reported that in two autopsies of COVID-19 patients, immunohistochemistry revealed a small number of CD3+ T lymphocytes infiltrating the alveolar septum, while CD20+ B-lymphocytes were rare. CD8+ T cells were slightly more prevalent than CD4+ T cells, and CD68 detection revealed a few macrophages. Some studies have suggested that the substantial decrease in the total number of lymphocytes in coronavirus patients may indicate that the virus consumes many immune cells and inhibits cellular immune function [82, 83].

After an antigen enters the body, the corresponding antigen-specific B cells are activated, induced to proliferate and eventually stimulated to differentiate into plasma cells. These plasma cells then produce specific antibodies that can enter the body fluid and exert immune effects. It is widely accepted that immunoglobulin M (IgM) provides the first line of defense during viral infections, prior to the generation of adaptive, high-affinity IgG responses that are important for long-term immunity and immunological memory [84]. Li et al. successfully developed a rapid detection IgG-IgM combined antibody test kit for the diagnosis of COVID-19. The kit has a sensitivity of 88.66% and a specificity of 90.63%, and can detect the infection within 15 minutes [85]. After the rehabilitation of most patients with the novel coronavirus, the body will produce specific antibodies that can kill and eliminate the virus.

On February 8, 2020, with the Pneumonia Diagnosis and Treatment Program for Novel Coronavirus Infection (Trial Version 5) [86] as a guide, The First People’s Hospital of Jiangxia District carried out the first phase of a new convalescent plasma treatment on three critically ill patients. After 12 to 24 hours of convalescent plasma therapy, the patients’ laboratory examination results, clinical signs and symptoms improved significantly. Plasma therapy not only is safe and potentially effective, but also stimulates humoral immunity [87].

Most COVID-19 patients have a good prognosis, while a few patients have mild symptoms in the early stage and suddenly deteriorate in the later stage of the disease or during the recovery process. A large number of patients have exhibited a ‘cytokine storm’ (the rapid production of cytokines such as tumor necrosis factor alpha, interleukin-1, interleukin-6 and interferon gamma) due to the viral infection, which sometimes has progressed to acute respiratory distress syndrome and multiple organ failure [12, 19]. It is already known that HCoV can spread from the respiratory tract to the central nervous system through transneuronal and hematogenous routes, resulting in encephalitis and neurological diseases [88]. The invasion of the blood-brain barrier by the coronavirus can destroy vascular endothelial connections, leading to blood-brain barrier dysfunction and enhanced permeability [89]. When the virus invades the human brain, it triggers immune damage, causing brain damage and acute or chronic inflammation, thus creating a vicious cycle.

#### Inflammation

Several current retrospective clinical studies have described COVID-19 patients with abnormally low lymphocyte counts, Prolonged prothrombin times and significantly increased lactate dehydrogenase levels. Patients transferred to the ICU had significantly higher white blood cell and neutrophil counts than those not transferred to the ICU, as well as higher levels of D-dimer, creatine kinase and creatine [20]. The complications in severe cases have included rhabdomyolysis, shock, acute cardiac injury and acute kidney injury. Several mechanisms are thought to link infections with acute vascular events, including the release of proinflammatory cytokines, the disruption of atherosclerotic plaques, physiological changes in the heart rate and vasoconstriction [90]. The inflammatory response in severe pneumonia is not limited to lung tissue; rather, the systemic inflammatory response is activated, and its amplification cascade impairs the function of distant organs [57, 91].

#### Hypercoagulability

Middle-aged and elderly patients account for the majority of COVID-19 patients (especially critically ill patients) with abnormally increased D-dimer levels, and such patients are more prone to embolic vascular events and cerebrovascular disease [20]. Umapathi et al. postulated that a hypercoagulable state predisposed a group of mainly critically ill SARS patients to large cerebral arterial thromboembolism [58]. Providers treating critically ill COVID-19 patients with underlying diseases such as hypertension, diabetes, cancer, etc. should be alert to the potential for hypercoagulability and regularly assess routine blood coagulation.

#### Ethics statement

Our research does not require an ethics statement.

## CONCLUSIONS

SARS-CoV-2 infection may involve the nervous system, and may cause diseases such as polyneuropathy, myopathy, cerebral infarction and central nervous system infections. Cerebral infarction is the second most common cause of death and the leading cause of adult disability worldwide. Patients with cerebrovascular diseases may face greater risks during infections, so it is necessary to strengthen protection to avoid infection, perform secondary prevention measures and monitor patients’ symptoms and vital signs. During the period of high incidence of COVID-19, neurologists need to pay great attention to the treatment of patients, especially those whose first symptoms are neurological symptoms
